# Examining Two Sets of Introgression Lines in Rice (*Oryza sativa* L.) Reveals Favorable Alleles that Improve Grain Zn and Fe Concentrations

**DOI:** 10.1371/journal.pone.0131846

**Published:** 2015-07-10

**Authors:** Qin Xu, Tian-Qing Zheng, Xia Hu, Li-Rui Cheng, Jian-Long Xu, Yu-Min Shi, Zhi-Kang Li

**Affiliations:** 1 Institute of Crop Sciences/National Key Facility for Crop Gene Resources and Genetic Improvement, Chinese Academy of Agricultural Sciences, 12 South Zhong-Guan-Cun Street, Beijing, China; 2 Institute of Agricultural Genomics at Shenzhen, Chinese Academy of Agricultural Sciences, Shenzhen, China; 3 Rice Research Institute, Guangxi Academy of Agricultural Sciences, Nanning, China; International Rice Research Institute, PHILIPPINES

## Abstract

In the modern world, the grain mineral concentration (GMC) in rice (*Oryza sativa* L.) not only includes important micronutrient elements such as iron (Fe) and zinc (Zn), but it also includes toxic heavy metal elements, especially cadmium (Cd) and lead (Pb). To date, the genetic mechanisms underlying the regulation of GMC, especially the genetic background and G × E effects of GMC, remain largely unknown. In this study, we adopted two sets of backcross introgression lines (BILs) derived from IR75862 (a Zn-dense rice variety) as the donor parent and two elite *indica* varieties, Ce258 and Zhongguangxiang1, as recurrent parents to detect QTL affecting GMC traits including Fe, Zn, Cd and Pb concentrations in two environments. We detected a total of 22 loci responsible for GMC traits, which are distributed on all 12 rice chromosomes except 5, 9 and 10. Six genetic overlap (GO) regions affecting multiple elements were found, in which most donor alleles had synergistic effects on GMC. Some toxic heavy metal-independent loci (such as *qFe1*, *qFe2* and *qZn12*) and some regions that have opposite genetic effects on micronutrient (Fe and Zn) and heavy metal element (Pb) concentrations (such as GO-IV) may be useful for marker-assisted biofortification breeding in rice. We discuss three important points affecting biofortification breeding efforts in rice, including correlations between different GMC traits, the genetic background effect and the G × E effect.

## Introduction

Minerals in cereal grains, especially iron (Fe) and zinc (Zn), are important sources of micronutrients for human health. Micronutrient malnutrition is still widespread in developing countries, especially among poor populations, whose daily caloric intake is mainly confined to staple cereals [1]. For example, in the developing countries of Asia, such as the Philippines and China, cereals provide approximately 50% of iron and zinc intake [2,3]. The number of people affected by zinc deficiency in these two countries is estimated to be 86 million and 10 million, respectively, with most living in rural areas [3] including southwestern China. Currently, the baseline concentrations of Fe and Zn in polished rice average 2.0 and 16.1 mg kg^-1^, respectively, while the target values are 14.5 and 24 mg kg^-1^, respectively, as proposed by the HarvestPlus Biofortification Breeding Program [4,5]. The development of biofortified cereal, especially mineral-dense rice, represents an efficient way to alleviate malnutrition in China and other developing countries [2,6].

On the other hand, the pollution of the arable lands by heavy metals, a side-effect of modern industry, has become increasingly severe; the levels of toxic minerals, especially cadmium (Cd) and lead (Pb), have been increasing in cereal grains, which threatens human health [7,8]. For example, Cd concentrations in soil samples collected in 2005 from Yangzhou, a developed area downstream of the Yangtze River in Jiangsu province, China, were significantly higher than those measured in the 1990s [9]. Moreover, in Taizhou, a developed area in Zhejiang province, the Cd and Pb concentrations in rice grains are approximately 8-fold and 4-fold greater, respectively, than those in other areas. Based on these data, the maximum daily intake of heavy metals by an adult in this area can reach 4.6-fold that of the Cd TDI (tolerable daily intake according to FAO/WHO) and twice that of the Pb TDI due to the contamination in rice grains [10] and not taking into account other foods produced on the same lands that are consumed daily, such as vegetables.

Rice, which has a relatively small genome, is by far the world’s most important staple food crop [11], and thus, both biofortification and reduction of heavy metal pollution in rice have attracted a great deal of attention. There are three important steps in the chain of mineral absorption from the soil to the plant: 1) root uptake, 2) xylem loading/root-to-shoot translocation and 3) phloem transport into the grain. All of these steps are associated with grain mineral concentration (GMC). Thus, GMC is a complex trait controlled by multiple quantitative loci. Few studies have taken a forward genetics approach to link different natural variations with GMC, although recently, some QTL mapping studies have been carried out using DH or RIL populations [12–16]. Attempts have also been made to identify the relationship between QTL and known transporters by in-silico mapping [17]. However, to date, despite the accumulating data from QTL mapping for GMC, only a few loci have been consistently identified in various studies. Three regions harboring QTL controlling Cd concentration in rice grain have been found on chromosomes 4, 7 and 11, respectively. Of these, only the QTL on chromosome 7 has been supported by evidence from four different tests, and it has been shown to represent a single gene, *OsNramp1* [18–23]. Multiple studies have revealed QTL related to Zn concentration on regions of chromosomes 5, 6 [16], 7 [12–14,16] and 8 [24–26].

G × E and genetic background effects are important for both QTL detection and breeding [27]. However, their effects on GMC traits have not been not fully explored, although molecular analysis has strongly suggested that differential transcription of the transporter gene *OsLCT1*, which is related to Cd, K, Mg, Ca and Mn levels, can cause huge phenotypic differences in *indica* and *japonica* rice [28]. Notably, although most reports focus on the effects of a few stably expressed loci on GMC traits, especially Cd concentration [15,19,21,23,29], in a RIL population, only 16.7% of the 12 QTL for five grain minerals (Cd, Cu, Fe, Zn and Mn) were stably expressed under field conditions in two nearby locations (only 32 km apart) [13]. This figure fell to 6.25% in tests carried out on a DH population in Southeastern China. Recently, in a study examining major QTL expressed in different environments in another DH population, only two out of nine major loci for GMC were found to overlap [26]. The genetic mechanisms underlying GMC in rice remain largely unclear, especially the genetic background effects and the possible genetic overlaps between different GMC traits.

The tropical *japonica* inbred line IR75862-206-2-8-3-B-B-B (here abbreviated as IR75862), which was developed by IRRI, is recommended for use as a zinc-dense donor [30]. However, the genetic mechanism underlying its high zinc levels, especially the genetic background and G × E effects of IR75862 and the possible correlations with other GMC traits, remains unknown. In this study, we used two sets of backcrossing inbred lines (BILs) derived from the same donor, IR75862, and two new elite varieties from Southwestern China, Ce258 and Zhongguangxiang1 (ZGX1), as recipients to assess the genetic background and G × E effects of GMC traits in rice by QTL mapping. The marker information obtained in this study and the elite lines may be useful for developing biofortified rice cultivars in the future.

## Materials and Methods

### Plant Materials and Field Experiments

Two sets of backcross recombinant-inbred line (BIL) populations were developed using IR75862-206-2-8-3-B-B-B (abbreviated as IR75862), a *japonica* waxy variety from International Rice Research Institute (IRRI) as the donor and two elite varieties, Ce258 and Zhongguangxiang1 (ZGX1) from Guangxi province, China, as recurrent parents.

A total of 401 BILs (with 201 and 200 lines in the Ce258 and ZGX1 backgrounds, respectively) in the BC_1_F_6_ generation and three parents, IR75862, Ce258 and ZGX1, were planted in two environments, i.e., Nanning (22.9° N, 108.3° E), Guangxi province, in 2009 (designated NN09) and Sanya (18.3° N, 109.3° E), Hainan province, in 2010 (designated SY10). Thirty-day-old seedlings were transplanted into paddy fields at the experimental stations of the Rice Research Institute, Guangxi Academy of Agricultural Sciences (GAAS) at Nanning and the Institute of Crop Sciences, Chinese Academy of Agricultural Sciences (CAAS) at Sanya. The BILs were arranged into two-row plots with 12 plants per row at a spacing of 20 × 17 cm with two replications. A randomized complete block design was used. At the maturing stage (approximately 40 days after flowering), seeds from each line were bulk-harvested, air-dried and stored for three months in a drying house before being evaluated for grain mineral concentration (GMC).

Basic physical and chemical properties of the soil in the paddy field were analyzed using routine analytical methods of agricultural chemistry [31]. The chemical and physical properties of the soil are shown in Table 1.

**Table 1:** Physical and chemical characteristics of the soil in the experimental fields.

Item	Nanning	Sanya
pH	9.74	8.51
Organic matter (g kg^-1^)	22.50	11.20
Total N (g kg^-1^)	10.98	7.10
Total P (g kg^-1^)	0.53	0.36
Total K (g kg^-1^)	26.45	28.15
Available N(mg kg^-1^)	104.81	89.34
Available P(mg kg^-1^)	38.52	32.45
Available K(mg kg^-1^)	133.34	28.29
Total Zn (mg kg^-1^)	1292.32	1107.31
Total Fe (mg kg^-1^)	2176.53	1992.60
Total Cd (mg kg^-1^)	0.22	0.19
Total Pb (mg kg^-1^)	23.45	24.17
Available Zn (mg kg^-1^)	26.54	20.03
Available Fe (mg kg^-1^)	218.81	216.63
Available Cd (mg kg^-1^)	0.15	0.11
Available Pb (mg kg^-1^)	18.72	20.09

### Evaluation of Grain Mineral Concentration (GMC)

Dried seeds from each line were de-hulled, polished and milled into flour according to the surging and grind-milling method reported by Jia et al. [32] to prevent mineral contamination, especially Fe. Approximately 0.3 g of rice flour was digested with 6 ml HNO_3_ and 0.2 ml H_2_O_2_ using a microwave digestion system (Microwave300, Anton PAAR, Graz, Austria). The settings were as follows: 700 W for 5 min, 700–1,200 W for 10 min and 1,200 W for 20 min. The samples were the transferred to a block heater at 160°C for further digestion. The remaining 1 ml of digested sample was diluted with 50 ml Milli-Q water prior to analysis.

Zn, Cd and Pb concentrations in digested samples were determined using inductively coupled plasma mass spectrometer (ICP-MS; Elan DRC-e, PerkinElmer, USA) in standard mode [31]. To determine total Fe concentration, an ICP-MS equipped with a dynamic reaction cell (DRC mode) pressurized with methane was used. For all determinations, calibration standard solutions were prepared by diluting 10 mg L^-1^ multi-element standard stock solution (PerkinElmer, USA) in 2% (v/v) HNO_3_; 10 μg L^-1^ mixtures of internal standard mix (PerkinElmer, USA) including scandium, germanium, yttrium and indium bismuth were used as internal standards, which were added to all samples, standards and blanks. Two standards and two controls were utilized in each sample batch. Three replicates were performed per sample.

### Genotyping and Map Construction

A total of 550 SSR markers as reported by Temnykh et al. [33] were used for the polymorphism assay. Of these, 129 and 133 markers showed polymorphism in the Ce258 and ZGX1 backgrounds, respectively. The linkage maps were constructed with Map Manager QTXb16 [34]; these maps cover 1,649.6 cM and 1,668.3 cM of the whole rice genome, with an average distance of 12.8 cM and 12.5 cM between adjacent markers, respectively.

### Data Analysis and QTL Mapping

Correlation analysis between the GMC traits of BILs in different environments and the comparison of mean values by analysis of variances (ANOVA) were conducted with SAS PROC CORR and GLM [35], respectively

A two-step analysis strategy was adopted to detect the associations between traits and markers. The first step of analysis involved classical one-way ANOVA in a SAS version 9.1 environment [35]. Phenotypic variance caused by each locus was compared with the residue variances. Since these two sets of BILs were suitable for traditional QTL mapping packages, the loci found to be significant were further confirmed using the built-in MET (multiple environments test) function in the QTL IciMapping v.3.3 package [36] to confirm the candidate marker regions identified by one-way ANOVA and to estimate the log likelihood ratio (LOD values) for both additive and genotype by environment (G × E) effects. The permutation method was used to obtain empirical thresholds for claiming QTL based on 1,000 runs of randomly shuffling the trait values [37], which ranged from LOD values of 2.8 for grain Fe concentration in Ce258-BILs at NN09 to 3.5 for grain Pb concentration in ZGX1-BILs at SY10. A locus found to be significant based on one-way ANOVA at a significance level of *P* ≤ 0.005 but insignificant based on the MET method at an empirical threshold was excluded as a false positive detection. To further examine the extent to which inconsistent QTL detection in different backgrounds actually arose from type II errors, all identified QTL in one genetic background were re-examined using the data from the other under a minimum threshold of LOD ≥ 1.2 [38]. Loci found to reach the minimum threshold in one set of BILs with supporting evidence that they were significant in the other set of BILs under the empirical threshold were listed, although the related parameters (LOD and additive values) were specifically indicated.

Mixed model are widely used in QTL analysis even for Q×E analysis [38]. However, as breeders pay more attention on stable QTL, here we used mixed model to combine phenotypic data in multiple environments to confirm reliable main-effect QTL affecting GMC by transforming the phenotypic data by the following model: Y^ijk^ = μ + Season_ij_ + Rep(Season)_ij_ + Line_k_ + e_ijk,_ where Y_ijk_ is the observed phenotype for the kth line in the jth replicate of the ith environment (season), μis the grand mean, Season_i_ is the random effect of the ith environment, Rep (Season)_ij_ is the random effect of the jth replicate in the ith environment, Line_k_ is the random effect of the kth line, and eijk is the error term, which follows an independent, identically distributed N (0, σ^2^) distribution. The MIXED procedure in SAS [35] was used to get the best linear unbiased estimate (BLUP) of the line effect, which was then added to the estimate of the grand mean. The loci passed this test in at least one background were also specifically indicated in the Results.

### Comparative Mapping and Pyramiding Effects Analysis

To compare the GMC QTL detected in this study with reported QTL or genes known to be associated with mineral density in rice, comparative mapping was carried out using previously reported procedures [39]. All markers or genes were tagged on the same reference sequence map, GRAMENE annotation sequence map 2009 [40], for the comparison. In combination with the genetic maps constructed in this study, QTL or genes allocated to the same chromosome bin were regarded as the same locus or tightly linked loci.

To analyze the pyramiding effects of introgressed donor alleles and allele combinations of the detected QTL for grain Zn and Fe concentrations in the Ce258-BILs, all BILs were grouped according to the allele and allele combinations at the QTL. Furthermore, Duncan’s t-test was employed to test the differences in grain Zn and Fe concentrations among different QTL combination groups.

## Results

### Performance of the Four GMC Traits in Parents and Their Descendants

The two recurrent parents, ZGX1 and Ce258, had significantly lower concentrations of Fe and Zn, especially Zn, than the donor parent, IR75862, in both environments (Table 2). ZGX1, Ce258 and IR75862 had similar Pb and Cd concentrations in both environments, except for Cd in NN09. Both sets of BILs exhibited transgressive segregation for the four traits, especially Fe and Zn concentrations, in both environments (S1 Fig).

**Table 2:** Phenotypic values of grain mineral concentrations detected in three parents and two sets of BILs in two environments (mg kg^-1^).

Trait	Environment ^1)^	Ce258 (P_1_)	ZGX1 (P_2_)	IR75862 (P_3_)	P_1_–P_3_ ^2)^	P_2_–P_3_	Ce258-BILs		ZGX1-BILs	
							Mean ± SD	CV%	Mean ± SD	CV%
Fe	NN09	1.692±0.056	1.473±0.306	3.106±0.012	-1.414	-1.633	1.041±0.581	55.77	1.235±0.392	31.73
	SY10	1.782±0.086	1.305±0.157	4.986±0.082	-3.204	-3.681 *	2.841±1.144	40.25	1.796±0.624	34.76
Zn	NN09	12.105 ± 0.295	14.848±0.133	26.794±0.345	-14.689 ***	-11.946 ***	13.917±2.371	17.04	11.266±1.027	9.12
	SY10	9.852 ± 0.102	11.947±0.183	24.710±0.001	-14.858 ***	-12.763 ***	12.539±1.863	14.86	11.237±1.337	11.90
Cd	NN09	0.045±0.001	0.136±0.001	0.144±0.001	-0.099 ***	-0.007	0.085±0.031	36.22	0.091±0.029	31.41
	SY10	0.041±0.005	0.034±0.001	0.049±0.001	-0.008	-0.005	0.011±0.011	41.53	0.011±0.005	43.84
Pb	NN09	0.010±0.004	0.011±0.001	0.017±0.001	-0.007	-0.006	0.023±0.004	19.10	0.021±0.009	43.61
	SY10	0.119±0.001	0.115±0.015	0.126±0.001	-0.011	-0.004	0.072±0.035	47.88	0.062±0.012	18.70

^1)^ NN09: 2009 summer season in Nanning, SY10: 2010 winter season in Sanya

^2)^ *, ** and *** represent significant differences at the P ≤ 0.05, 0.01 and 0.001 levels, respectively.

### Correlations among the Four GMC Traits in Two Sets of BILs

As shown in Table 3, significant correlations (in **bold**, *P* < 0.05) were found between different GMC traits in BILs in the same environment. However, little correlation was found between the same traits in different environments, which strongly indicates that there were environmental effects on the GMC traits. For example, significant positive correlations (both 0.40) were found between grain Zn and Fe concentrations in Ce258 background BILs in both environments, although the positive correlations between Zn and Cd were also significant (0.42 and 0.28, respectively). There was also positive correlation (0.57) between Cd and Pb in the ZGX1 background in the SY10 environment.

**Table 3:** Correlation coefficients between grain mineral concentrations in backcross inbred lines with two genetic backgrounds, Ce258 (below the diagonal) and ZGX (above the diagonal) measured in 2009 in Nanning (NN09) and in 2010 in Sanya (SY10).

		NN09				SY10			
		Fe	Zn	Cd	Pb	Fe	Zn	Cd	Pb
NN09	Fe		**0.20** *	**0.21** *	**-0.19** *	-0.09	-0.10	-0.04	-0.02
	Zn	**0.40** ****		0.07	**0.33** ****	-0.04	-0.01	-0.13	0.01
	Cd	**0.25** **	**0.42** ****		-0.12	**-0.23** **	-0.08	-0.14	0.07
	Pb	**0.26** **	**0.26** **	0.09		-0.08	**-0.20** *	0.06	0.00
SY10	Fe	0.03	-0.03	-0.06	-0.06		0.27**	0.07	0.09
	Zn	-0.02	0.04	0.02	0.07	**0.40** ****		**0.28** **	0.11
	Cd	0.09	-0.13	0.00	**-0.41** ****	**0.34** ****	**0.28** **		**0.57** ****
	Pb	**0.34** ****	-0.21*	-0.03	**-0.53** ****	-0.05	**-0.22** **	0.10	

Coefficients showing the correlations between the same trait in different seasons and between different traits in the same season are listed; *, **, *** and **** represent significance levels of P ≤ 0.05, 0.01, 0.001 and 0.0001, respectively.

### Identification of Background-independent (BI) and/or Stably Expressed Loci Controlling the Four GMC Traits in Two Sets of BILs

**Fig 1:**
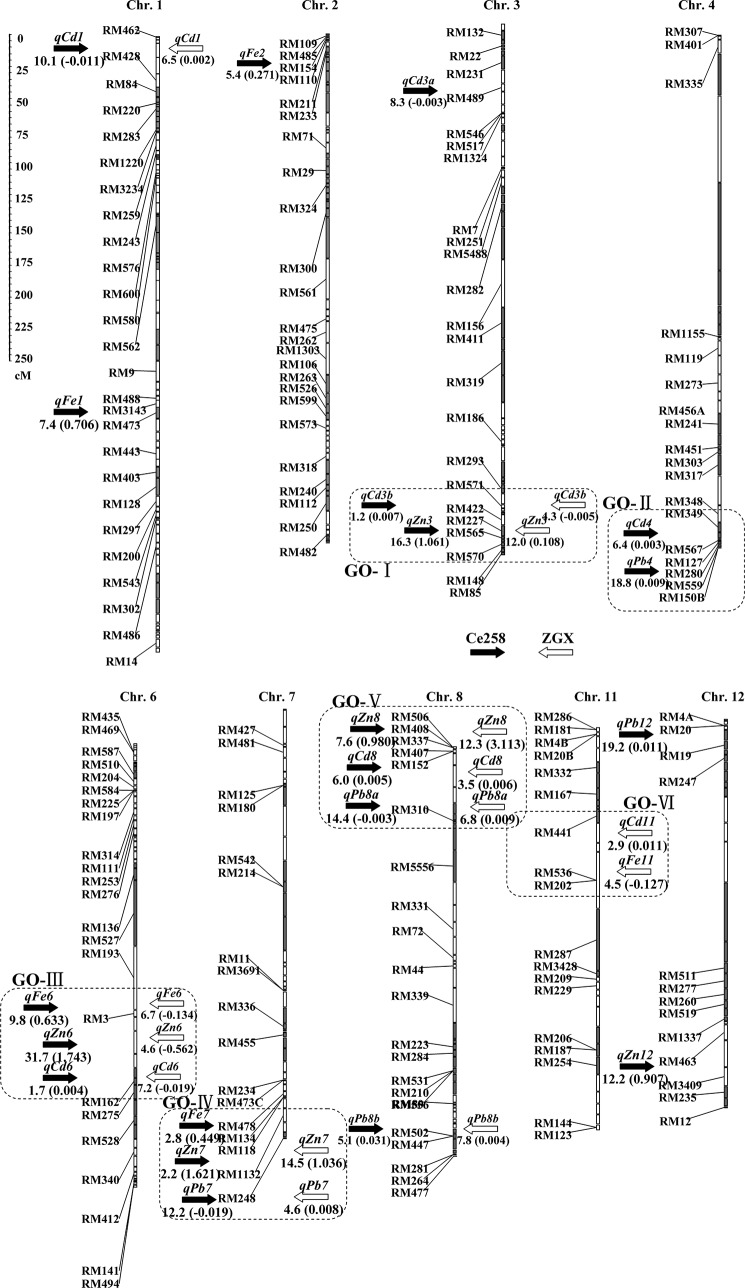
The integrated genetic map and distribution of QTL affecting grain mineral concentration (GMC) of Fe, Zn, Cd and Pb detected in the two sets of backcross introgression lines (BILs) derived from IR75862, a Zn dense variety as donor parent and two elite *indica* varieties, Ce258 and Zhongguangxiang1 as recurrent parents. QTL on the left of the chromosomes show those detected in BILs of Ce258 × IR75862 whereas those on the right of the chromosomes in BILs of Zhongguangxiang1 × IR75862. Digits on the left and inside brackets under QTL bars represent LOD value and additive effect (in 10^3^ mg kg^-1^) of QTL. Dotted line box stands for the genetic overlap regions affecting GMC of different mineral elements.

As shown in Table 4 and Fig 1, a total of 22 loci were detected in two sets of BILs, which were distributed across the genome except for chromosomes 5, 9 and 10. The averaged LOD scores were 9.9 (ranging from 1.2 to 31.7) and 7.0 (ranging from 2.9 to 14.5) for additive effects in the Ce258 and ZGX1 backgrounds, respectively. Four (*qFe6*, *qFe7*, *qZn8* and *qCd6*) and one (*qCd11*) loci were found to possess significant G × E effects in the Ce258 and ZGX1 backgrounds, respectively.

**Table 4:** QTL detected in the two backcross introgression populations (Ce258-BILs and ZGX1-BILs) in 2009 in Nanning (NN09) and in 2010 in Sanya (SY10). LOD(A) and LOD(AE) indicate LOD values for additive effects and additive by environment effects, respectively; PVE(A) and PVE(AE), phenotypic variances explained by additive effects and additive by environment effects, respectively; A, AE1 and AE2 indicate the additive effects and the additive by environment effects detected at NN09 and SY10, respectively.

				Ce258–BILs							ZGX1–BILs							Previously reported QTL or genes ^5)^
Trait	QTL ^1)^	Chr.	Marker region ^2)^	LOD ^3)^(A)	LOD(AE)	PVE(A)	PVE(AE)	A ^4)^	AE1	AE2	LOD(A)	LOD(AE)	PVE(A)	PVE(AE)	A	AE1	AE2	
Fe	***qFe1*** #	1	RM302–RM486	7.4		8.0		0.706										*OsYSL18* [44]
	*qFe2*	2	RM154–RM211	5.4		7.5		0.271										*qFe2–1* [24]
	***qFe6*** #	6	RM3–RM340	9.8	5.0	18.3	10.9	0.633	0.315	–0.338	6.7		10.2		–0.134			*qFe6* [26]
	*qFe7* #	7	RM134–RM1132	2.8	9.9	6.3	16.7	0.449	–0.114	0.114								
	*qFe11* &	11	RM441–RM202								4.5		5.5		–0.127			
Zn	*qZn3* #	3	RM293–RM85	16.3		14.4		1.061			12.0		11.1		0.108			
	***qZn6*** #	6	RM3–RM340	31.7		24.8		1.743			4.6		7.3		–0.562			
	*qZn7* #	7	RM134–RM1132	2.2		2.0		1.621			14.5		7.0		1.036			*LOC_Os07g43040* [17]
	***qZn8*** #	8	RM407–RM152	7.6	8.4	18.0	24.9	0.980	1.151	–1.151	12.3		11.2		3.113			*qZn8–1* [24]; *qZn8b* [26]; *qZn8* [25]
	***qZn12*** #	12	RM1337–RM3409	12.2		12.2		0.907										*qZn12–1* [24]
Cd	***qCd1*** #	1	RM462–RM428	10.1		7.2		–0.011			6.5		4.1		0.002			
	*qCd3a*	3	RM1324–RM489	8.3		11.2		–0.003										Segment_on_Chr3_for_Cd [[29]; *OsNRAMP2* [45]; *OsARD2* [46]
	*qCd3b* # ^,^ &	3	RM293–RM227	1.2		1.1		0.007			4.3		5.2		–0.005			*qCdc3* [15]
	*qCd4*	4	RM348–RM280	6.4		8.8		0.003										*qCd4–2* [49]; *OsZIP3* [[50]
	*qCd6* ^#^	6	RM136–RM3	1.7	9.4	1.7	2.6	0.004	–0.007	0.007	7.2		9.9		–0.019			Segment_on_Chr6_for_Cd [29]; *OsLCT1* [41]
	***qCd8*** #	8	RM506–RM407	6.0		7.5		0.005			3.5		3.4		0.006			Segment_on_Chr8_for_Cd [29]
	*qCd11* &	11	RM332–RM441								2.9	2.6	1.7	0.7	0.011	0.009	–0.009	*qCCBR–11a* [51]; *LOC_Os11g07980* [17]
Pb	*qPb4* #	4	RM241–RM280	18.8		8.8		0.009										*OsYSL13*, *OsYSL16* [47]; *OsYSL12* [48]
	***qPb7*** #	7	RM336–RM134	12.2		35.1		–0.019			4.6		4.5		0.008			*LOC_Os07g43040* [17]
	*qPb8a* &	8	RM408–RM310	14.4		13.2		–0.003			6.8		7.2		0.009			
	***qPb8b*** &	8	RM80–RM447	5.1		4.9		0.031			7.8		5.9		0.004			
	*qPb12*	12	RM20–RM4A	19.2		16.5		0.011										*qPb12–2* [14]

QTL with symbols ‘#’ and ‘&’ are those stably detected in both the NN09 and SY10 environments in the Ce258 and ZGX1 backgrounds, respectively. Loci that passed the main-effect QTL confirmation test (See step 3 in the Materials and Methods) in at least one background are shown in **bold**.

Underlined markers are those closer to putative QTL.

Underlined numbers are the parameters of the QTL detected under the sub-threshold of 1.2 ≤ LOD ≤ the threshold estimated by permutation test as described in the Materials and Methods.

Additive effects resulting from the substitution of ZGX1 or Ce258 alleles by IR75862 alleles

Numbers in brackets are reference codes, as listed in the reference section.

Five QTL (*qFe1*, *qFe2*, *qFe6*, *qFe7* and *qFe11*) were found to be associated with grain Fe concentration, totally explaining 67.7% (including 27.6% by G × E interaction) and 15.7% of the phenotypic variance, with an average absolute value for additive effect of 0.515 mg kg^-1^ (ranging from 0.271 to 0.706) and 0.131 mg kg^-1^ (ranging from 0.127 to 0.134) in the Ce258 and ZGX1 background, respectively (Table 4 and Fig 1). The IR75862 alleles at all loci (except *qFe6* and *qFe11* in the ZGX1 background) increased Fe concentrations. One QTL (*qFe6*) was consistently detected, with the maximum LOD scores (9.8 and 6.7) for additive effects in opposite directions in both backgrounds, suggesting that this QTL is associated with the BI-locus. QTL *qFe6* and *qFe7* were found to have significant G × E effects in the Ce258 background.

Five QTL (*qZn3*, *qZn6*, *qZn7*, *qZn8* and *qZn12*) were identified for grain Zn concentration, totally explaining 96.3% (including 24.9% by G × E interaction) and 36.3% of the phenotypic variance, with an average absolute value for additive effect of 1.262 mg kg^-1^ (ranging from 0.907 to 1.743) and 1.205 mg kg^-1^ (ranging from 0.108 to 3.113) in Ce258-BILs and ZGX1-BILs, respectively (Table 4 and Fig 1). The IR75862 alleles at all loci (except *qZn6* in the ZGX1 background) increased Zn concentrations. Four BI-loci (*qZn3*, *qZn6*, *qZn7* and *qZn8*) were consistently detected in both backgrounds, but only *qZn8* had significant a G × E effect, with a LOD score of 8.4 in the Ce258 background.

A total of seven loci (*qCd1*, *qCd3a*, *qCd3b*, *qCd4*, *qCd6*, *qCd8* and *qCd11*) were found to be associated with grain Cd concentration (Table 4 and Fig 1). These loci together explained 37.5% and 24.3% of the phenotypic variance, with an average absolute additive effect of 0.006 mg kg^-1^ (ranging from 0.003 to 0.011) and 0.009 mg kg^-1^ (ranging from 0.002 to 0.019) in the Ce258 and ZGX1 backgrounds, respectively. The IR75862 alleles at *qCd3b*, *qCd4*, *qCd6* and *qCd8* in the Ce258 background and *qCd1*, *qCd8* and *qCd11* in the ZGX1 background increased Cd concentrations, while those at *qCd1* and *qCd3a* in the Ce258 background and *qCd3b* and *qCd6* in the ZGX1 background had the opposite effect. Four BI-loci (*qCd1*, *qCd3b*, *qCd6* and *qCd8*) were consistently detected in both backgrounds, with opposite directions of genetic effects, except for *qCd8*. In addition, *qCd6* and *qCd11* were found to have G × E effects in the Ce258 and ZGX1 backgrounds, respectively.

Five Pb loci (*qPb4*, *qPb7*, *qPb8a*, *qPb8b* and *qPb12*) were identified for grain Pb concentration, totally explaining 78.5% and 17.6% of the phenotypic variance, with an average absolute additive effect of 0.015 mg kg^-1^ (ranging from 0.003 to 0.019) and 0.007 mg kg^-1^ (ranging from 0.004 to 0.009) in the Ce258 and ZGX1 backgrounds, respectively (Table 4 and Fig 1). The IR75862 alleles at all loci (except *qPb7* and *qPb8a* in the Ce258 background) increased Pb concentrations. Three BI-loci (*qPb7*, *qPb8a* and *qPb8b*) were consistently detected in both backgrounds, with opposite directions of genetic effects, except for *qPb8b*. No QTL was detected for G × E effect in both backgrounds.

In total, 12 of the 22 (54.5%) QTL were found to be expressed in both genetic backgrounds and were therefore regarded as background independent loci (BI-loci). Of the 22 loci, 14 (63.6%) and five (22.7%) were stably detected in both environments in the Ce258 and ZGX1 background, respectively. All of these loci were therefore regarded as environment independent loci or stably expressed loci (SE-loci; Table 4). Among these, only one locus (*qCd3b*) was stably expressed in both backgrounds in both environments. Interestingly, all BI-loci were also stably expressed across environments.

### Genetic Overlaps Underlying the Correlation among GMC Traits

There was significant genetic overlap found between loci affecting different GMC traits (Fig 1). A total of six regions (GO-I–VI) distributed on chromosomes 3, 4, 6, 7, 8 and 11 were responsible for genetic overlap for different GMC (Fig 1). The first region (GO-I), which includes two loci, *qZn3* and *qCd3b*, is located on chromosome 3 (indicated by RM293). The second region (GO-II), containing *qCd4* and *qPb4*, is located on chromosome 4 (indicated by RM280). The third region (GO-III), containing three loci (*qFe6*, *qZn6* and *qCd6*), is located near marker RM3 on chromosome 6. The fourth region (GO-IV), which includes three loci (*qFe7*, *qZn7* and *qPb7*), is associated with RM134 on chromosome 7. The fifth region (GO-V), harboring three loci (*qZn8*, *qCd8* and *qPb8a*), is linked to RM407 on chromosome 8. Finally, the sixth region (GO-VI), harboring two loci (*qFe11* and *qCd11*), is located on chromosome 11 (indicated by RM441).

Synergistic effects were found for the IR75862 alleles for all four GMC traits in all GO regions except for the following: GO-I and GO-VI in the ZGX1 background and GO-IV and GO-V in the Ce258 background. Specifically, the IR75862 alleles in GO-IV increased Zn and Fe levels but decreased Pb levels in the Ce258 background.

### Effects of the Introgressed Donor Alleles on GMC Traits in Both Backgrounds

Higher grain Zn concentrations were found for the donor parent IR75862 compared to the two recipients, Ce258 and ZGX1, as shown in Table 2, and almost all favorable alleles at the QTL affecting Zn concentration identified in the two BILs were derived from IR75862 (Table 4). Three QTL (*qZn3*, *qZn7* and *qZn8*) were identified as BI-loci, and the introgressed IR75862 alleles at these QTL increased Zn concentrations in both backgrounds (Table 4). At NN09 and SY10, three and two types of BILs in the Ce258 background exhibited significantly higher Zn concentrations in the grain, respectively, compared with the recurrent parent (Table 5). At NN09, the line with pyramiding of three IR75862 alleles at three loci (*qZn6* + *qZn7* + *qZn12*) behaved the best (23.9 mg kg^-1^), approaching the level of the donor parent (26.8 mg kg^-1^). BILs with different two-allele combinations (*qZn7* + *qZn8* and *qZn7* + *qZn12*) had the second highest levels, i.e., 19.4 and 17.8 mg kg^-1^, respectively. The Zn concentrations of the BILs with introgression of IR75862 alleles at *qZn6* + *qZn8*, *qZn3*, *qZn8*, *qZn6*, *qZn7* and *qZn12* were only marginally higher than that of the recurrent parent. At SY10, the pyramiding effects of the two combinations (*qZn6* + *qZn12* or *qZn8* + *qZn12*) behaved the best (18.1 and 15.0 mg kg^-1^, respectively). However, the pyramiding effects of Fe-QTL alleles were not as significant as those of Zn-QTL alleles in the Ce258 background, and no significant pyramiding effect was detected for the Zn- and Fe-QTL alleles in the ZGX1 background. These results strongly suggest that Zn concentration could be significantly improved by pyramiding non-allelic Zn-QTL alleles, although the suitable allele combinations for different genetic backgrounds and environments should also be taken into consideration.

**Table 5:** Pyramiding effects of IR75862 alleles on grain Zn concentrations in the Ce258 background in Nanning (NN09) and Sanya (SY10).

	Ce258 Background					ZGX1 Background				
Types ^1)^		Grain Zn Con. (mg/kg)(Mean ± SD) ^3)^		Grain Fe Con. (mg/kg)(Mean ± SD)			Grain Zn Con. (mg/kg)(Mean ± SD) ^3)^		Grain Fe Con. (mg/kg)(Mean ± SD)	
	N ^2)^	NN09	SY10	NN09	SY10	N	NN09	SY10	NN09	SY10
DP	2	**26.8 ± 0.3** ^**a**^	24.7 ± 0.0 ^a^	3.1 ± 0.0 ^a^	5.0 ± 0.1 ^a^	2	26.8 ± 0.3 ^a^	24.7 ± 0.0 ^a^	3.1 ± 0.0 ^a^	5.0 ± 0.1 ^a^
*qZn6*+*qZn7*+*qZn12*	2	**23.9 ± 0.1** ^**a**^		1.9 ± 0.5 ^abcd^						
*qZn7*+ *qZn8*	2	**19.4 ± 0.3** ^**b**^		2.8 ± 0.0 ^ab^						
*qZn7*+*qZn12*	2	**17.8 ± 1.7** ^**bc**^		2.2 ± 0.6 ^abc^						
*qZn6*+*qZn12*	6		**18.1 ± 1.1** ^**b**^		3.3 ± 1.4 ^ab^					
*qZn8*+*qZn12*	2		**15.0 ± 1.2** ^**c**^		3.0 ± 0.4 ^ab^					
*qZn6*+*qZn8*	4	16.2 ± 3.5 ^bcd^		1.3 ± 0.5 ^cd^				10.6 ± 1.4 ^c^		1.6 ± 0.2 ^b^
*qZn3*	2	14.5 ± 1.2 ^cd^	11.4 ± 1.8 ^de^	0.8 ± 0.0 ^d^	2.5 ± 0.1 ^ab^	6	11.0 ± 1.4 ^c^	11.2 ± 1.3 ^c^	1.3 ± 0.7 ^b^	1.8 ± 0.6 ^b^
*qZn8*	21	14.8 ± 3.8 ^cd^	11.4 ± 1.2 ^de^	1.1 ± 0.5 ^cd^	2.7 ± 0.8 ^ab^	2	12.2 ± 0.9 ^c^	10.6 ± 0.7 ^c^	0.8 ± 0.1 ^b^	1.4 ± 0.0 ^b^
*qZn6*	12	14.0 ± 2.0 ^cd^	13.0 ± 3.3 ^bcde^	0.9 ± 0.5 ^cd^	4.6 ± 6.8 ^ab^	24	10.5 ± 1.2 ^c^	11.8 ± 1.5 ^c^	1.3 ± 1.2 ^b^	1.6 ± 0.4 ^b^
*qZn7*	2	14.5 ± 0.7 ^cd^	13.3 ± 2.8 ^bcde^	1.6 ± 0.5 ^bcd^	2.7 ± 0.3 ^ab^					
*qZn12*	12	13.9 ± 1.1 ^cd^	13.4 ± 1.4 ^bcde^	0.7 ± 0.2 ^d^	2.7 ± 0.3 ^ab^					
RP	2	12.1 ± 0.3 ^d^	9.9 ± 0.1 ^de^	1.7 ± 0.1 ^bcd^	1.8 ± 0.1 ^b^	2	14.8 ± 0.1 ^b^	11.9 ± 0.2 ^c^	1.5 ± 0.3 ^b^	1.3 ± 0.2 ^b^

DP = Donor parent, RP = Recurrent parent, referring to RP1 in the t-tests for the Ce258 background and RP2 for the ZGX1 background, respectively.

N = observations for the t-test, which equals the number of lines times two replications.

Mean values with the same letter are not significantly different (P ≤ 0.05).

## Discussion

### Effect of Genetic Background on QTL Detection for GMC

Accumulating evidence indicates that the genetic background effect is unavoidable during the genetic improvement of favorable GMC in rice. For example, the differential expression of *OsLCT1*, a major gene, controls the different Cd translocation abilities between *indica* and *japonica* rice, even though the *OsLCT1* alleles of *indica* and *japonica* rice are almost identical [28,41].

In the current experiment, only 12 (54.5%) of the 22 GMC loci were expressed in both the Ce258 and ZGX1 backgrounds. Eight of the 10 genetic background-specific loci were expressed in the Ce258 background and the other two were expressed in the ZGX1 background. When we consider different traits, grain Fe concentration is the top trait affected by the genetic background effect. Four of the five Fe loci were background specific, with three loci in Ce258 and one in ZGX1. Grain Cd and Pb concentrations were also affected by genetic background. Three of the seven Cd loci were specifically expressed (two in Ce258 and one in ZGX1), and two of the Pb loci were specifically expressed in the Ce258 background. Relatively speaking, grain Zn concentration is the least affected by genetic background. Of the five Zn loci, only one (*qZn12*) was specifically expressed in the Ce258 background. Interestingly, when we examined the allelic effects by loci, we found that most alleles from IR75862 at the same BI locus tended to have opposite effects in the Ce258 and ZGX1 backgrounds. For example, at the GO-III region (including *qFe6*, *qZn6* and *qCd6*) on chromosome 6 (indicated by RM3), the additive effects for IR75862 alleles for GMC traits were all positive in the Ce258 background but negative in the ZGX1 background (Table 4). The same is true for *qCd3b*, *qCd1*, *qPb7* and *qPB8a*. Furthermore, it is much easier to identify elite lines (better than the recurrent parent) with relatively high Zn concentrations in the Ce258 background than in the ZGX1 background (Table 5), although similar numbers of lines with favorable IR75862 allele introgression for Zn were found (data not shown). Therefore, when QTL information is applied to marker-assisted breeding for biofortification of rice, the genetic background effects on QTL expression and the direction of genetic effects should be noted.

### Effect of the Environment on QTL Detection for GMC

The effects of the environment on GMC in crops are still largely unclear. In wheat, GMC traits are largely affected by environmental factors, and Cd and Zn traits have relatively high heritability, whereas Fe has the strongest G × E effects [42,43]. However, Fe- and Zn-dense varieties of bean and rice exhibit relatively stable behavior under normal conditions [2], while the QTL mapping results from a DH rice population for Ca, Fe, K, Mg, Mn, P and Zn show that GMC QTL are largely environment-dependent [26].

In the current study, we also investigated the G × E effect for each GMC locus. Surprisingly, additive effects other than the G × E effects played more important roles in the phenotypic variances in these two sets of BILs. This result also differs from our first impression of the very low correlations between the same GMC in different environments in the same background (Table 2), which may largely be due to inheritable factors other than G × E interactions. We also found that for all GMC traits, a total of 14 and five loci were stably expressed in both environments in the Ce258 and ZGX1 backgrounds, respectively. Notably, the pyramiding lines in the Ce258 background had higher grain Zn concentrations than the recurrent parent (Table 5), and different environments appeared to favor different combinations of IR75862 alleles, which may represent another reflection of genetic background and G × E effects. For example, the combination of IR75862 alleles at *qZn6* + *qZn7* + *qZn12* appears to perform the best for breeders in Western China (Nanning), while the combination *qZn6* + *qZn12* or *qZn8* + *qZn12* would be a better choice for breeders in Southern China (Sanya). Breeders involved in marker-assisted rice biofortification breeding should also pay attention to the effect of the environment on allele combinations at different QTL.

### Comparison of Identified GMC QTL with Those Reported in Earlier Studies

Comparative mapping was carried out according to a reference sequence map, GRAMENE annotation sequence map 2009 [40], to compare the GMC QTL detected in this study with previously reported QTL or genes known to be associated with mineral density in rice. Of the 13 regions harboring 22 QTL for GMC variations identified in the two sets of IR75862-BILs, 11 (84.6%) were found to cover known QTL or functional genes for GMC in rice. For instance, *qFe1* (with flanking markers RM302 and RM486 on chromosome 1) was mapped to an adjacent region to that harboring *OsYSL18*, which encodes an iron (III)-deoxymugineic acid transporter specifically expressed in the reproductive stage in rice [44]. QTL *qFe2* (associated with RM211 on chromosome 2) was mapped to the region containing *qFe2-1* [24]. QTL *qCd3a* (flanked by markers RM1324 and RM489 on chromosome 3) covers the region containing a QTL for Cd [29] and two genes, i.e., *OsNRAMP2*, encoding a membrane transporter for metal ions [45] and *OsARD2*, which is associated with high grain Zn concentrations [46]. QTL *qCd3b* in GO-I (flanked by markers RM293 and RM227 on chromosome 3) was mapped to the region containing *qCd3* [15]. Both *qPb4* and *qCd4*, which are associated with RM280 in GO-II on chromosome 4, were mapped to the region containing three clustered genes, *OsYSL13*, *OsYSL16* [47] and *OsYSL12* [48], which are associated with Fe or Zn concentrations, along with the Cd-associated QTL *qCd4-2* [49] and Zn transporter gene *OsZIP3* [50]. QTL *qCd6* and *qFe6* (associated with RM3 in GO-III on chromosome 6) were mapped to a region harboring a QTL for Cd [29], the low-affinity cation transporter gene *OsLCT1* [41] and an Fe-associated QTL, *qFe6* [26]. QTL *qZn7* and *qPb7* (linked with RM134 in GO-IV on chromosome 7) were mapped to the region containing gene *LOC_Os07g43040*, which is associated with grain Fe and Zn contents [17]. QTL *qZn8* and *qCd8* (linked to RM407 in GO-V on chromosome 8) were mapped to the region containing with QTL *qZn8-1*[24], *qZn8b* [26] and *qZn8* [25] and a Cd-related QTL [29]. QTL *qCd11* (with flanking markers RM332 and RM441 in GO-VI on chromosome 11) was mapped to the adjacent region with QTL *qCCBR-11a* [51] and *LOC_Os11g07980*, which is involved in Cd contents [17]. QTL *qZn12* and *qPb12* (associated with RM1337 and RM20 on chromosome 12) were mapped to the region harboring *qZn12-1* [24] and *qPb12-2* [14], respectively. QTL regions for the GMC traits mentioned above that were identified in different mapping populations and diverse environments could be beneficial for MAS-based development of biofortified rice cultivars. Allelism of the above GMC QTL identified in this study to the reported GMC-related genes will need to be further clarified after fine mapping and cloning of the GMC QTL.

### Implications for Rice Biofortification Breeding

The results of this study have three useful implications for the development for biofortified cultivars. First, it is well known that as positive ions that are mostly in valence state two, the minerals Fe, Zn, Cd and Pb are thought to be absorbed by higher plants in competition mode, with no highly specific transporters [52]. Negative correlations have been reported between the concentrations of certain ions (such as Fe, Mn and Ni versus Cd/Fe and Mn/Ni versus Pb) under polluted paddy field conditions [53], and some released cultivars with a combination of low brown rice Cd and Pb levels but high micronutrient levels were preliminarily identified by Li et al. [53]. However, under common paddy field conditions without heavy pollution, we found that in the two sets of BILs examined, with a common zinc-dense donor but two different *indica* cultivar backgrounds, significant correlations between different GMC traits were mostly positive, except for the correlations between Pb in NN09 and two heavy metals (Cd and Pb) in SY10 in the Ce258 background (Table 3). This type of positive correlation was also observed in a previous study under normal paddy field conditions with no heavy pollution [24]. The correlation observed in the current study could be partially explained by the presence of six GO regions containing loci affecting more than one GMC, where a synergistic mode other than the reverse mode of the effect of the IR75862 allele on different GMC traits may have played a major role (Table 4). Thus, when performing biofortification breeding in cereals, a possible trade-off between high micronutrient levels and increased ability for heavy metal accumulation should be fully taken into consideration. The linkage drag for different GMC traits should be considered when applying the Fe or Zn loci in these six GO regions (Table 4). In addition, fine mapping studies are required for further application of the favorable loci/alleles for the improvement of micronutrient levels in rice by MAS.

Second, there are also two GO regions (IV and I) covering loci that may be useful for biofortification breeding. Of these, the IR75862 alleles in the GO-IV region may be the best choice for breeders because they simultaneously increased Fe and Zn levels but decreased Pb levels in the Ce258 background. Another choice would be the GO-I region, where the IR75862 alleles increased Zn levels but decreased Cd levels in the ZGX1 background.

Third, pyramiding breeding is a powerful technique for improving micronutrient levels, as indicated by the grain Zn concentrations in the Ce258 background (Table 5). Therefore, marker-assisted co-introgression of the IR75862 alleles at heavy metal-independent loci, such as *qFe1/2* and *qZn12*, or the IR75862 alleles at these loci with opposite additive effects on micronutrient and heavy metal elements, e.g., *qFe7* and *qZn7* (GO-IV), represents a new, feasible strategy for increasing the Fe and/or Zn concentrations in rice grains. Additionally, the main-effect loci for GMC, especially those shown in bold in Table 4, e.g., *qFe1*, *qFe6*, *qZn6*, *qZn8* and *qZn12*, will be highly useful. Finally, loci that may play important roles in the pyramiding effects but have not been confirmed under a relatively stringent threshold (such as *qZn7*) should not be neglected in breeding practices.

## Conclusion

A total of 22 loci responsible for GMC traits in rice were identified. Among these, *qFe6*, *qZn6*, *qZn8*, *QCd1*, *qCd8*, *qPb7* and *qPb8b* were consistently detected in different backgrounds and environments. Six genetic overlap (GO) regions affecting multiple elements were identified, in which most donor alleles exhibited synergistic effects on GMC.

Detection of QTL for GMC was strongly influenced by genetic background, and the donor alleles at some loci functioned in opposite manners in different backgrounds. Therefore, attention should be paid to the genetic background effects on QTL expression and the direction of genetic effects when QTL information is applied to marker-assisted breeding for biofortification in rice. We found that the effects of the environment on QTL detection and allele combinations were significant and should therefore also be considered during marker-assisted biofortification breeding in rice.

Synergistic mode may play a major role in the relationships among different GMCs. To avoid side effects from high levels of micronutrients and heavy metals, some toxic heavy metal-independent loci such as *qFe1*, *qFe2* and *qZn12*, and some regions such as GO-IV, with opposite genetic effects on micronutrient and heavy metal levels, should take priority in marker-assisted biofortification breeding in rice.

Pyramiding breeding is a promising strategy for GMC improvement, especially for grain Zn concentration. However, the pyramiding effects of different alleles appear to be dependent on background and environment. The relatively insignificant pyramiding effect on grain Fe concentrations could be largely due to inheritable factors.

## Supporting Information

S1 Fig Distributions of the GMC traits in two sets of BILs and their parents under two environments. NN09 = Nanning (22.9° N, 108.3° E), Guangxi province, in 2009; SY10 = Sanya (18.3° N, 109.3° E), Hainan province, in 2010. The trait values of Ce258 and Zhongguangxiang1 (ZGX1) in different environments were also indicated by arrows of the same colors as that for the population.
(TIF)

## References

[1] Bhullar NK, Gruissem W Nutritional enhancement of rice for human health: the contribution of biotechnology. Biotechnol Adv.2013; 31: 50–57. 10.1016/j.biotechadv.2012.02.001 22343216

[2] Gregorio GB, Htut T. Micronutrient-dense rice: developing breeding tools at IRRI In: Mew TW, Brar DS, Peng S, Dawe D, Hardy B, editors. Rice science: innovations and impact for livelihood; Beijing, China International Rice Research Institute, Chinese Academy of Engineering, Chinese Academy of Agricultural Sciences;2003: pp. 1022.

[3] Ma G, Jin Y, Li Y, Zhai F, Kok FJ, Jacobsen E, et al Iron and zinc deficiencies in China: what is a feasible and cost-effective strategy? Public Health Nutr.2008; 11: 632–638. 17894916 10.1017/S1368980007001085

[4] Pfeiffer WH, McClafferty B Biofortification: Breeding Micronutrient-Dense Crops Breeding Major Food Staples: Blackwell Publishing Ltd;2008 pp. 61–91.

[5] Biofortification Progress Briefs: HarvestPlus;2014.Available:http://www.harvestplus.org/.

[6] De Steur H, Gellynck X, Blancquaert D, Lambert W, Van Der Straeten D, Qaim M Potential impact and cost-effectiveness of multi-biofortified rice in China. N Biotechnol.2012; 29: 432–442. 10.1016/j.nbt.2011.11.012 22154941

[7] Al-Saleh I, Shinwari N Report on the levels of cadmium, lead, and mercury in imported rice grain samples. Biol Trace Elem Res.2001; 83: 91–96. 11694006 10.1385/BTER:83:1:91

[8] Hang X, Wang H, Zhou J, Ma C, Du C, Chen X Risk assessment of potentially toxic element pollution in soils and rice (*Oryza sativa*) in a typical area of the Yangtze River Delta. Environ Pollut.2009; 157: 2542–2549. 10.1016/j.envpol.2009.03.002 19344985

[9] Huang SS, Liao QL, Hua M, Wu XM, Bi KS, Yan CY, et al Survey of heavy metal pollution and assessment of agricultural soil in Yangzhong district, Jiangsu Province, China. Chemosphere.2007; 67: 2148–2155. 17275882 10.1016/j.chemosphere.2006.12.043

[10] Fu J, Zhou Q, Liu J, Liu W, Wang T, Zhang Q, et al High levels of heavy metals in rice (*Oryza sativa* L.) from a typical E-waste recycling area in southeast China and its potential risk to human health. Chemosphere.2008; 71: 1269–1275. 10.1016/j.chemosphere.2007.11.065 18289635

[11] Dawe D, Pandey S, Nelson A Emerging trends and spatial patterns of rice production In: Pandey S, Byerlee D, Dawe D, Dobermann A, Mohanty S, Rozelle S et al, editors. Rice in the global economy: strategic research and policy issues for food security. Los Banos: International Rice Research Institute;2010 pp. 15–36.

[12] Lu KY, Li LZ, Zheng XF, Zhang ZH, Mou TM, Hu ZL Quantitative trait loci controlling Cu, Ca, Zn, Mn and Fe content in rice grains. Journal of Genetics.2008; 87: 305–310. 19147920 10.1007/s12041-008-0049-8

[13] Shen XH, Cao LY, Shao GS, Zhan XD, Chen SG, Wu WM, et al QTL mapping for the content of five trace elements in brown rice. Molecular Plant Breeding.2008; 6: 1061–1067.

[14] Tang SQ QTL mapping for cooking and nutrient quality traits of rice Hangzhou: Zhejiang University;2007.

[15] Zhang X, Zhang G, Guo L, Wang H, Zeng D, Dong G, et al Identification of quantitative trait loci for Cd and Zn concentrations of brown rice grown in Cd-polluted soils. Euphytica.2011; 180: 173–179.

[16] Zhong L QTL analysis on mineral elements content in rice Chengdu: Sichuan Agricultural University;2010.

[17] Chandel G, Samuel P, Dubey M, Meena R In silico expression analysis of QTL specific candidate genes for grain micronutrient (Fe/Zn) content using ESTs and MPSS signature analysis in rice (*Oryza sativa* L.). Journal of Plant Genetics and Transgenics.2011; 2: 11–22.

[18] Ishikawa S, Abe T, Kuramata M, Yamaguchi M, Ando T, Yamamoto T, et al A major quantitative trait locus for increasing cadmiumspecific concentration in rice grain is located on the short arm of chromosome 7. Journal of Experimental Botany.2010; 61: 923–934. 10.1093/jxb/erp360 20022924 PMC2814118

[19] Abe T, Taguchi-Shiobara F, Kojima Y, Ebitani T, Kuramata M, Yamamoto T, et al Detection of a QTL for accumulating Cd in rice that enables efficient Cd phytoextraction from soil. Breeding Science.2011; 61: 43–51.

[20] Ueno D, Kono I, Yokosho K, Ando T, Yano M, Ma JF A major quantitative trait locus controlling cadmium translocation in rice (*Oryza sativa*). New Phytol.2009; 182: 644–653. 10.1111/j.1469-8137.2009.02784.x 19309445

[21] Ueno D, Koyama E, Kono I, Ando T, Yano M, Ma JF Identification of a novel major quantitative trait locus controlling distribution of Cd between roots and shoots in rice. Plant and Cell Physiology.2009; 50: 2223–2233. 10.1093/pcp/pcp160 19884249

[22] Ueno D, Yamaji N, Kono I, Huang CF, Ando T, Yano M, et al Gene limiting cadmium accumulation in rice. Proceedings of the National Academy of Sciences.2010; 107: 16500–16505. 10.1073/pnas.1005396107 PMC2944702 20823253

[23] Tezuka K, Miyadate H, Katou K, Kodama I, Matsumoto S, Kawamoto T, et al A single recessive gene controls cadmium translocation in the cadmium hyperaccumulating rice cultivar Cho-Ko-Koku. Theoretical Applied Genetics.2010; 120: 1175–1182. 10.1007/s00122-009-1244-6 20039013

[24] Garcia-Oliveira AL, Tan L, Fu Y, Sun C Genetic identification of quantitative trait loci for contents of mineral nutrients in rice grain. Journal of Integrative Plant Biology.2009; 51: 84–92. 10.1111/j.1744-7909.2008.00730.x 19166498

[25] Bekele BD, Naveen GK, Rakhi S, Shashidhar HE Genetic evaluation of recombinant inbred lines of rice (*Oryza sativa* L.) for grain zinc concentrations, yield related traits and identification of associated SSR markers. Pak J Biol Sci.2013; 16: 1714–1721. 24506038 10.3923/pjbs.2013.1714.1721

[26] Du J, Zeng D, Wang B, Qian Q, Zheng S, Ling HQ Environmental effects on mineral accumulation in rice grains and identification of ecological specific QTLs. Environ Geochem Health.2013; 35: 161–170. 10.1007/s10653-012-9473-z 22760687

[27] Li ZK, Yu SB, Lafitte HR, Huang N, Courtois B, Hittalmani S, et al QTL x environment interactions in rice. I. heading date and plant height. Theor Appl Genet.2003; 108: 141–153. 12961067 10.1007/s00122-003-1401-2

[28] Uraguchi S, Kamiya T, Clemens S, Fujiwara T Characterization of OsLCT1, a cadmium transporter from indica rice (*Oryza sativa*). Physiol Plant.2014. 10.1111/ppl.12189 24627964

[29] Ishikawa S, Ae N, Yano M Chromosomal regions with quantitative trait loci controlling cadmium concentration in brown rice(*Oryza sativa*). New Physiologist.2005; 168: 345–350. 10.1111/j.1469-8137.2005.01516.x 16219074

[30] Impa SM, Morete MJ, Ismail AM, Schulin R, Johnson-Beebout SE Zn uptake, translocation and grain Zn loading in rice (*Oryza sativa* L.) genotypes selected for Zn deficiency tolerance and high grain Zn. J Exp Bot.2013; 64: 2739–2751. 10.1093/jxb/ert118 23698631 PMC3697949

[31] Lu RK Analytical methods of soil and agricultural chemistry Beijing: China Agricultural Science and Technology Press;1999.

[32] Jia Q, Xu Q, Shi Y-M, Hu X, Sun Y, Cheng L-R, et al A robust and cost-effective sgoc method for testing rice iron concentration in biofortified breeding. Acta Agronomica Sinica.2010; 36: 979–987.

[33] Temnykh S, Park WD, Ayres N, Cartinhour S, Hauck N, Lipovich L, et al Mapping and genome organization of microsatellite sequences in rice (*Oryza sativa* L.). TheorApplGenet.2000; 100: 697–712.

[34] Manly KF, Cudmore JRH, Meer JM Map Manager QTX, cross-platform software for genetic mapping. Mammalian Genome.2001; 12: 930–932. 11707780 10.1007/s00335-001-1016-3

[35] Inc. SASI SAS/STAT 9.1 user's guide. Cary, North Carolina.: SAS Institute Inc.;2004.

[36] Li H, Ribaut J-M, Li Z, Wang J Inclusive composite interval mapping (ICIM) for digenic epistasis of quantitative traits in biparental populations. TAG Theoretical and Applied Genetics.2008; 116: 243–260. 17985112 10.1007/s00122-007-0663-5

[37] Churchill GA, Doerge RW Empirical threshold values for quantitative trait mapping. Genetics.1994; 138: 963–971. 7851788 10.1093/genetics/138.3.963 PMC1206241

[38] Malosetti M, Ribaut J-M, van Eeuwijk FA The statistical analysis of multi-environment data: modeling genotype-by-environment interaction and its genetic basis. Frontiers in Physiology.2013; 4. 10.3389/fphys.2013.00044 PMC3594989 23487515

[39] Zheng TQ, Wang Y, Ali AJ, Zhu LH, Sun Y, Zhai HQ, et al Genetic effects of background-independent loci for grain weight and shape identified using advanced reciprocal introgression lines from Lemont/Teqing in rice (*Oryza sativa* L.). Crop Science.2011; 51: 2025–2034.

[40] cMap.Gramene database. Release 43#; 2014; http://www.gramene.org/.

[41] Uraguchi S, Kamiya T, Sakamoto T, Kasai K, Sato Y, Nagamura Y, et al Low-affinity cation transporter (OsLCT1) regulates cadmium transport into rice grains. Proceedings of the National Academy of Sciences.2011; 108: 20959–20964. 10.1073/pnas.1116531109 PMC3248505 22160725

[42] Gomez-Becerra H, Yazici A, Ozturk L, Budak H, Peleg Z, Morgounov A, et al Genetic variation and environmental stability of grain mineral nutrient concentrations in Triticum dicoccoides under five environments. Euphytica.2010; 171: 39–52.

[43] McLaughlin MJ, Parker DR, Clarke JM Metals and micronutrients—food safety issues. Field Crops Research.1999; 60: 143–163.

[44] Aoyama T, Kobayashi T, Takahashi M, Nagasaka S, Usuda K, Kakei Y, et al OsYSL18 is a rice iron(III)-deoxymugineic acid transporter specifically expressed in reproductive organs and phloem of lamina joints. Plant Mol Biol.2009; 70: 681–692. 10.1007/s11103-009-9500-3 19468840 PMC2706380

[45] Belouchi A, Kwan T, Gros P Cloning and characterization of the OsNramp family from *Oryza sativa*, a new family of membrane proteins possibly implicated in the transport of metal ions. Plant Mol Biol.1997; 33: 1085–1092. 9154989 10.1023/a:1005723304911

[46] Anuradha K, Agarwal S, Rao YV, Rao KV, Viraktamath BC, Sarla N Mapping QTLs and candidate genes for iron and zinc concentrations in unpolished rice of MadhukarxSwarna RILs. Gene.2012; 508: 233–240. 10.1016/j.gene.2012.07.054 22964359

[47] Narayanan NN, Vasconcelos MW, Grusak MA Expression profiling of *Oryza sativa* metal homeostasis genes in different rice cultivars using a cDNA macroarray. Plant Physiol Biochem.2007; 45: 277–286. 17468002 10.1016/j.plaphy.2007.03.021

[48] Banerjee S, Chandel G Understanding the role of metal homeostasis related candidate genes in Fe/Zn uptake, transport and redistribution in rice using semi-quantitative RT-PCR. J Plant Mol Biol Biotechnol.2011; 2: 33–46.

[49] Kashiwagi T, Shindoh K, Hirotsu N, Ishimaru K Evidence for separate translocation pathways in determining cadmium accumulation in grain and aerial plant parts in rice. BMC Plant Biology.2009; 9: 8 10.1186/1471-2229-9-8 19154618 PMC2632998

[50] Ramesh SA, Shin R, Eide DJ, Schachtman DP Differential metal selectivity and gene expression of two zinc transporters from rice. Plant Physiology.2003; 133: 126–134. 12970480 10.1104/pp.103.026815 PMC196588

[51] Chen ZD, Wang ZF, He JB, Zhong WG, Wang J, Yang J, et al Mapping of QTLs of Cd2+ content in brown rice under Cd2+ stress in rice. Hereditas (Beijing).2009; 31: 1135–1140. 10.3724/sp.j.1005.2009.01135 19933095

[52] Clarkson DT, Hanson JB The mineral nutrition of higher plants. Annual Review of Plant Physiology.1980; 31: 239–298.

[53] Li B, Wang X, Qi X, Huang L, Ye Z Identification of rice cultivars with low brown rice mixed cadmium and lead contents and their interactions with the micronutrients iron, zinc, nickel and manganese. Journal of Environmental Sciences.2012; 24: 1790–1798. 10.1016/s1001-0742(11)60972-8 23520849

